# Synthesis, Morphology, and Biomedical Applications of Plasma-Based Polymers: Recent Trends and Advances

**DOI:** 10.3390/polym16192701

**Published:** 2024-09-24

**Authors:** Mohammad Mizanur Rahman Khan, Shakila Parveen Asrafali, Thirukumaran Periyasamy

**Affiliations:** 1 Department of Mechanical Engineering, Gachon University, 1342 Seongnam-daero, Sujeong-gu, Seongnam-si 13120, Gyeonggi-do, Republic of Korea; mmrkhan@gachon.ac.kr; 2 Department of Fiber System Engineering, Yeungnam University, 280 Daehak-Ro, Gyeongsan 38541, Gyeongbuk, Republic of Korea

**Keywords:** plasma-based polymers, plasma, synthesis, morphology, biomedical applications

## Abstract

The achievement of large-scale applications of plasma-based polymers in biomedical sectors does not satisfy the appropriate level although a substantial amount of research is already performed. In this context, further investigations are necessary to design and synthesize plasma polymers for biomedical applications. Among the polymeric materials, plasma-based polymers have attracted substantial attention owing to their numerous advantages like faster processing, lower costs, eco-friendly waste, biocompatibility, and versatility, making them excellent materials for biomedical applications. Further, polymer synthesis using plasma polymerization techniques can avoid the time-consuming conventional multistep synthesis procedure. Plasma polymerization also offers a significant solution to overcome the numerous difficulties in the traditional approach where polymers can be directly attached to the desired surface using a plasma process, without disturbing the growing chain, and, thus, prevent an additional process such as grafting. Nevertheless, the design of appropriate plasma-based synthesis methods, optimization of the plasma process parameters, and exploration of polymer-based biocompatibility approaches are still challenging research areas. Regarding the surface morphological features of these plasma polymers, they possess several characteristics, such as wettability, adhesion capacity, and so on, that are important considerations in biomedical applications. In this review, numerous recent approaches to plasma polymerization methods along with different precursor phases used for such kind of synthesis of polymeric materials are discussed. The morphological aspect of the synthesized plasma polymers connected with biomedical applications is also reported in this review. Finally, promising aspects of plasma polymers for biomedical applications are briefly reported in this work. This review may offer an extensive reference for upcoming perceptions of plasma-based polymers and their applications in biomedical sectors.

## 1. Introduction

Plasma is matter that consists of ionized gas in equilibrium or nonequilibrium situations. Plasmas comprise many states of electrons, ions, atoms, radicals, and molecules. In addition, they emit UV and visible light. In free space, plasma that has the identical density of negatively charged particles (electrons) and positively charged particles (ions) behaves neutrally [1,2,3]. Numerous technologies have already been developed based on plasma, leading to surface alteration processes, including etching and managing chemical moieties. Thus, research based on plasma-based technologies has led to many large-scale industrial applications [4]. Plasma-enhanced technologies are also applied, to a large degree, to modify the characteristics of biological materials and their fabrications where plasma-generated species can act as catalysts for the optimizations of the chemical properties of materials, including surface properties [5,6,7,8]. In this connection, plasma-based polymers are usually employed in various biomedical applications [9]. In a typical plasma polymerization synthesis process, the required monomers are usually reactive chemical compounds. But plasma polymerization does not require a reactive chemical species like monomers; instead, plasma can initiate the polymerization reaction [9]. Concerning material processing, low-pressure plasmas are typically used for polymer synthesis and modification.

Among the plasma-based polymer synthesis, the focus on using an atmospheric-pressure plasma (APP) approach with specific application processes can be found in very few review reports in the literature. N. Bondt et al. [10] reported the plasma-based polymer synthesis using an electrical discharge process is considered one of the first reports on plasma polymerization. The first applications of the synthesized plasma polymers were reported in Ref. [11]. Further studies on enhancing material properties through plasma-based polymers were vigorously carried out, emphasizing the interaction between plasma and different substances [12,13,14,15]. Nowadays, plasma-based polymer synthesis is popular owing to the numerous antimicrobial or biomaterial applications [16,17,18], use in electrical devices [19,20,21], and surface modification [22,23,24].

**Figure 1:**
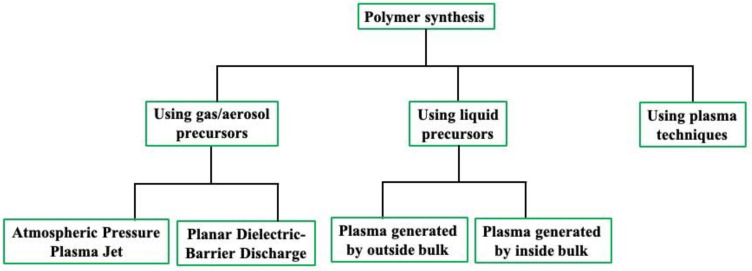
Numerous synthesis approaches of plasma-based polymerization.

**Figure 2:**
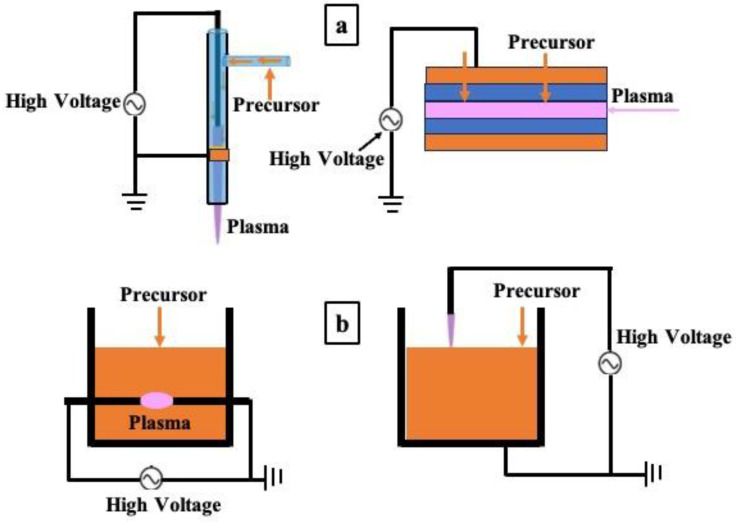
Schematic illustration of the synthesis approach using plasma: (**a**) gas/aerosol-through-plasma approach (left: jet type, right: dielectric-barrier discharge form) and (**b**) solution plasma technique (left: in-solution plasma, right: on-solution plasma) [3].

Plasma can be produced artificially using lasers, waves, flames, nuclear reactions, etc. However, the ubiquitous method of plasma initiation is the electrical discharge process originating from electric power sources [25,26]. The plasma produced by electric power is referred to as “nonequilibrium plasma” because heavy particles, such as ions, have a different temperature (or kinetic energy) than electrons. This plasma generation using electric power is known as ‘cold plasma’ or ‘non-thermal plasma’ [26]. Such a cold plasma polymerization approach comprises comparatively low temperatures of the heavy particles, which can avoid the damage to the substrates. This method is useful for several reasons, such as the generation of free radicals from precursors, high concentrations of reactive species, the ability to optimize the insolubility problems of materials, and the capacity to reduce the necessity of oxidant or reductant in the synthesis process [26,27,28,29,30,31]. Moreover, plasma polymerization also offers fast processing, high reactivity, low cost, green synthesis, and so on [32,33]. Depending on the necessary plasma operating pressure, the non-thermal plasmas are broadly divided into vacuum plasma and APP. The conditions required for plasma generation and the characteristics of plasma-based polymer synthesis are reported in Refs. [22,32,33,34,35,36]. Such reports will help further investigations of plasma polymerization methods and their applications in various biomedical sectors. The numerous synthesis techniques of the plasma polymerization are presented in Figure 1, and the schematic outline depending on the employed precursors of the plasma-based polymerization approaches is shown in Figure 2 [3,37,38].

The surface morphology of the synthesized polymers is also a vital concern in their biomedical applications. For example, R. Seeböck et al. [39] reported the modifications of surface topography (for example, surface roughening) of the polyimide. K.L. Mittal reported that the physical surface roughening plays a substantial role in the improvement of adhesion in organic polymers [40]. Moreover, the synthesis process of polymeric materials using plasma polymerization, their morphology, and their application in biomedical sectors are also a vital concern for researchers. In this context, numerous studies can be found in the literature in which the research topic is intended for biomedical applications of plasma polymers [9]. In this review, the biomedical applications of such plasma-based polymers and related phenomena are briefly discussed. 

This review aims to present a summary of current research on the plasma-based polymer synthesis processes using different approaches. Furthermore, the numerous polymer synthesis methods based on the precursor phase used are also discussed. The morphological aspect of the synthesized plasma polymers is also reported in this study as such morphological topography is still a challenging area for large-scale applications. Finally, the present review intends to provide the idea and promising aspects of these plasma polymers for biomedical applications.

## 2. Synthesis Approaches Used for Polymer Synthesis Using Plasma

Numerous synthesis approaches to plasma polymerization are reported by the researchers as presented in Figure 1 [3,41,42,43,44,45,46,47,48,49,50,51,52,53,54,55,56,57,58,59]. Synthesis techniques are classified according to the precursors utilized, plasma techniques used, etc., in the polymerization process. In the following sections, we will review the details of such synthesis techniques of plasma polymerizations.

### 2.1. Polymer Synthesis Using Gas/Aerosol-Type Precursors

In the synthesis of polymers using gas/aerosol-type precursors, first, a discharge gas is needed to generate “atmospheric pressure plasma”. Aerosol or gaseous-type precursors are used in this type of polymerization process (Figure 2a). In this case, materials that exist in a gaseous state at room temperature are utilized as precursors and liquid-state materials are mostly used with plasmas that act as aerosols through atomization with gas. This type of method is typical for the accumulation of polymer films [41,42,43,44,45,46,47,48,49,50,51,52,53,54,55,56,57,58,59]. Actually, the “Atmospheric Pressure Plasma” synthesis technique involves two types, subject to the phase of the applied precursor. The first category of this type of polymerization is the use of gas or aerosol as a precursor [17,60], and the second type is the use of liquid/solution [3], as displayed in Figure 2a,b.

#### 2.1.1. Atmospheric Pressure Plasma Jet Method

An “Atmospheric Pressure Plasma Jet” used for the polymerization process is a device that can generate directional plasma from a narrow nozzle. In this process, gas flow occurs with high input energy. Further, local processing is made possible by polymerization with atmospheric pressure plasma jet devices since the process region is constrained to the size of the jet plasma plume [41,44]. In this technique, precursors like gas/aerosol are fragmented by passing through the area of plasma generation. Afterward, fragmented precursors become neutral, or they can recombine elsewhere in the plasma stream, which is finally accumulated on a substrate. Such substrate lies outside the plasma area [61].

In this context, pin–ring electrodes comprising different metals such as Al and Cu were used in the atmospheric pressure plasma jet method for the synthesis of polymers by the researchers [41,42,46]. For instance, Zhang et al. [41] applied an “Atmospheric Pressure Plasma Jet” for the coating on rough surfaces using poly(methyl methacrylate) (PMMA) with the pin electrode (Cu rod). In this case, the plasma discharge gas was argon, which was introduced over the domain of the quartz body in an aerosol state. The authors reported the use of alternating current (AC) as a power generation source in the range of 10 kHz to 60 kHz with the highest voltage of 17 kV delivered by the pin electrode [41]. Pandiyaraj et al. [42]. reported the use of “Atmospheric Pressure Plasma Jet” for polymer synthesis where both the pin and ring electrodes were Cu. The author claimed that the AC power was provided with the highest voltage of 40 kV, current of 30 mA, and frequency of 50 kHz to generate the “Atmospheric Pressure Plasma”. Doherty et al. [46] synthesized polystyrene from heptylamine using a particular powered ring electrode and He gas for plasma discharge. The monomer heptylamine in the aerosol state was bubbled by the He gas for this polymerization process. The applied power was a sinusoidal current using a voltage of 8 kVp–p and a frequency of 10 kHz. 

**Figure 3:**
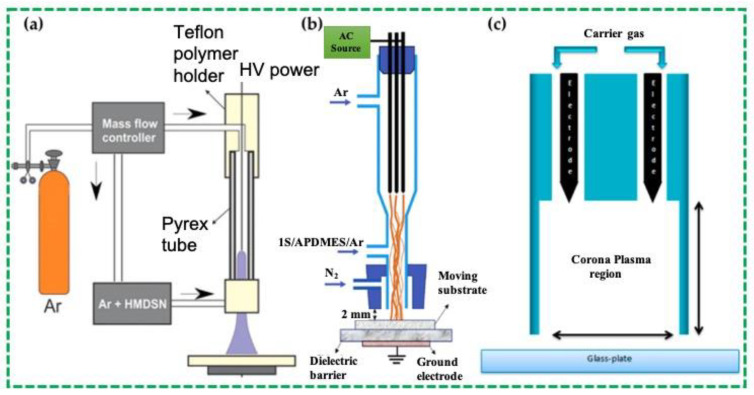
Structures of different types of pin electrodes in the “Atmospheric Pressure Plasma Jet” as reported in Ref. [3]. (**a**) Polymerization of HMDSN without using a grounded ring electrode [62], (**b**) Polymerization TMS and APDMES on glass [43], and (**c**) Generation of He corona plasma using two pin electrodes [63], Copyright 2021, MDPI.

Simple pin-type electrodes were used for the generation of “Atmospheric Pressure Plasma Jet” by researchers, as reported in Refs. [43,62,63]. This type of arrangement for the polymerization process is shown in Figure 3. Kodaira et al. [62] reported the investigation of the polymerization of hexamethyldisilane (HMDSN) by “Atmospheric Pressure Plasma” without using a grounded ring electrode (Figure 3a). The authors applied a voltage of 12 kVp–p, and the discharge gas and the monomer carrier gas were Ar [62].

The “Atmospheric Pressure Plasma” polymerization tetramethylsilane (TMS) and 3- aminopropyl(diethoxy) methylsilane (APDMES) were reported for the superhydrophobic coating purposes on glass [43]. In this process, three-pin electrodes were used to generate “Atmospheric Pressure Plasma”. These electrodes comprised stainless steel needles, which were situated at 120° interims in a glass reactor. In this case, a mixture of TMS and APDMES was used as a precursor, which was bubbled with Ar gas. This type of plasma process was operated by the AC power at a frequency of 11.5 kHz to create an “Atmospheric Pressure Plasma” discharge. 

Three array-type jets and an exceptional kind of shielding system have been reported and explained in Refs. [44,45,64,65], and this technique is called the “guide-tube and bluff-body (GB) system”. The authors claim that with the help of this approach, the area of high-density plasma can be expanded more than 60 times [65]. The significance of this synthesis approach is that it is possible to synthesize a copolymer [59], a pin-hole-free polymer [45], a conducting polymer [64], and a single-crystalline polymer [65] successfully. A conducting polymer and a transparent polymer were synthesized by Kim et al. [47] utilizing a pin-type “Atmospheric Pressure Plasma Jet” with the GB system. The experimental arrangements, as explained by the authors, involved applying a wide glass tube as the guide tube and polytetrafluoroethylene support as the bluff body. The plasma was generated with the help of a tungsten wire electrode. For the polymerization process, the sinusoidal power with a voltage of 4–5 kV and a frequency of 30 kHz were utilized via the tungsten wire electrode where a diffused-glow plasma was formed. Commercial “Atmospheric Pressure Plasma Jet” devices were used to polymerize hexamethyldisiloxane (HMDSO) [48,49,50]. For this polymerization, the plasma was generated by applying a frequency of 19 kHz and a plasma voltage of 285 V. The discharge gases were N_2_, O_2_, and air in this case. Moosburger-Will et al. reported that such “Atmospheric Pressure Plasma Jet” method was useful for in-line processing [66].

In summary, the “Atmospheric Pressure Plasma Jet” approach has numerous advantages as reported by the scientists. Such advantages and/or particularity depend on the applied precursors and instrumental techniques, especially on the power sources used for plasma generation. The particularity of such a process includes the enhancement of the flashover functioning of a polypropylene surface, easy deposition of polymer film, copolymerization, PMMA film deposition, polymerization of PMMA, etc. The important precursors utilized for this approach were methyl-methacrylate (MMA), heptylamine, acetylene, aniline, pyrrole, tetramethylsilane (TMS), etc. For this approach, the plasma was produced by employing an AC (pulse sine) power in the range of 2–2.8 W, a frequency of 11.5–50 kHz, and a voltage of 2–40 kV. The used discharge gases for each of the processes were Ar, He, N_2_, O_2_, and air. Although numerous advantages have been reported utilizing this approach, it is still a challenging area to design the appropriate precursors, power, and discharge gases for the plasma-based polymerization process to attain the expected large-scale industrial applications. The proper precursor with a suitable instrumental design and adjustable power sources can enhance the performance of this “Atmospheric Pressure Plasma Jet Method”.

#### 2.1.2. Dielectric-Barrier Discharge Method

**Figure 4:**
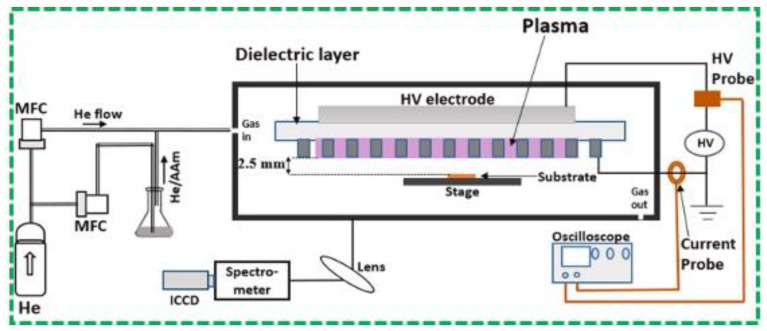
Schematic representation of experimental setup displaying the plasma source side view, electrical, spectrometer, and camera arrangement [67]. Reproduced with permission from Ref. [67]. Copyright 2021, Elsevier.

Researchers’ contributions to the polymerization processes include the functionalization of different types of polymer coatings using the planar dielectric-barrier discharge (DBD) method [67]. For example, to attain amine-functionalized polymer coatings, an atmospheric pressure surface dielectric-barrier discharge (SDBD) was produced in the He gas by Jumal Ibrahim et al. [67]. The authors investigated the electrical and optical properties of the surface plasma-produced SDBD comprising a mesh electrode, along with its applications in cell therapy. The schematic form of the experimental setup that was employed for this investigation is presented in Figure 4. A sinusoidal high-voltage source that could deliver 1 to 40 kVpk-pk at a frequency of 20 to 70 kHz was used to run the SDBD. Using a mass flow controller, the He gas was utilized as a process gas and presented into the area near the plasma source at a flow rate of 8 standard Lmin^−1^. At applied voltages ranging from 3.5 kV to 5 kVpk-pk, the plasma device may produce glow-like discharge structures that are steady over the electrode region according to current-voltage measurements and increased charge-coupled device (ICCD) camera images [67].

Regarding the planar-type DBD, the used electrodes are planar and are concealed by dielectric material [68,69]. The intention to use covered electrodes is to avoid possible arc formation from them. Plasma forms between the electrodes when one side of the electrode receives a high-voltage current. The advantages of using this method are that it is simpler to cover a larger area than with the “Atmospheric Pressure Plasma Jet” method since the size of the electrodes determines the size of the plasma area.

For example, polymer deposition utilizing a planar DBD approach with permanent electrodes is reported in references [51,52,68,69,70]. To enhance the antifouling characteristics of low-density polyethylene (LDPE) films through the copolymerization process, a plasma generator is used by Pandivaraj et al. [68]. In this process, plasma was produced by AC power with a voltage of 40 kV and a frequency of 50 Hz by the powered electrode. Ramkumar et al. [69] also used the same method where the authors utilized PEG methyl ether methacrylate (PEGMA) as a precursor to increase the biocompatibility of LDPE films. In addition, the diffuse coplanar surface barrier discharge (DCSBD) technique was adopted in reference [70], where the authors showed that the thin-layer plasma was produced on DCSBD surfaces using a high-voltage sine wave. 

The adjustable upper electrode was utilized by the researchers to enhance the deposition uniformity [53,54,71]. For instance, the mechanical properties of the generated coating were improved by Bardon et al. [54] by utilizing DBD plasma. In this case, the plasma was produced with the use of AC power of 110 W with a peak-to-peak voltage of 11 kV [54]. Manakhov et al. [71] also used the same technique for the copolymerization of maleic anhydride (MA) and C_2_H_2_ for carboxyl-rich coatings in a metallic cube chamber. Patterned hydrophilic/phobic coatings were deposited on any material surfaces utilizing acrylic acid and propargyl methacrylate by DBD approach [55]. The authors used Ar for both the purpose of discharge gas and precursor carrier gas for this process. DBD plasma polymerization was employed by Jalaber et al. [72] to create environmentally friendly, catalyst-free polymer synthesis. The plasma was created by passing a 10 kHz sinusoidal voltage between two plane-parallel electrodes. The dopamine acrylamide precursor was atomized with a nebulizer [72]. The plasma-initiated chemical vapor deposition (PCVD) was employed for the polymerization process in reference [57,58,59]. This technique involved igniting the DBD plasma with an ultra-short square-wave pulse power to deposit a polymer layer with a high degree of polymerization.

In summary, the use of the Dielectric-Barrier Discharge (DBD) Method for the polymerization process has numerous advantages, as reported by the researchers, where the authors used different types of precursor and plasma sources. The significance and particularity (for example, enhancement of biocompatibility, surface hydrophilization) are varied for the synthesis process depending on the nature of precursors and used plasma sources. Among them, the significance and particularity of each approach depending on the applied precursor and plasma sources for the process include the enhancement of antifouling properties, biocompatibility, surface hydrophilization, improvement of mechanical properties, copolymerization, age-resistant coatings, hydrophilicity, etc. Examples of utilized precursors for these processes are acrylic acid (AA) and poly (ethylene glycol) (PEG), poly (ethylene glycol) methyl ether methacrylate (PEGMA), etc. It is noteworthy that different gases like Ar, N_2_, He, and O_2_ were used for the plasma sources. In most of the cases, plasma was generated with the use of AC power of 3.3–110 W with a peak-to-peak voltage of 4–15 kV and at a frequency of 4–70 kHz. Focus can be provided on the use of precursor plasma sources and techniques of the process to get better performance of the polymerization process which can be useful for large-scale applications.

### 2.2. Polymer Synthesis Using Liquid-Type Precursors

**Figure 5:**
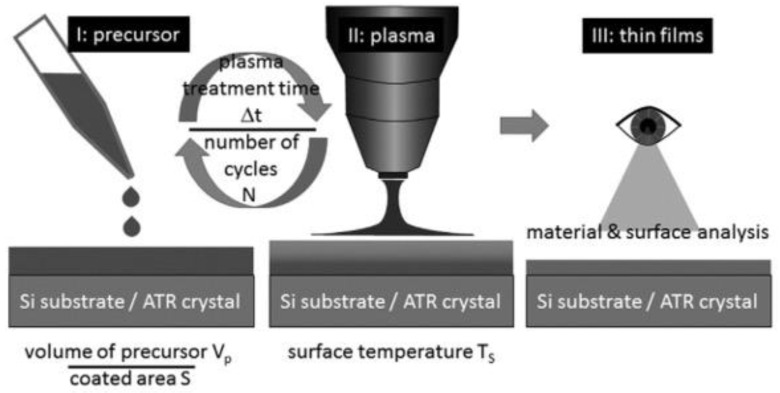
Different process steps of liquid-assisted plasma enhanced chemical vapor deposition (LA-PECVD). Applied process parameters are as follows: laboratory temperature 25 °C, humidity 30%, surface temperature (T_S_) ≈ 25 °C, S = 26 mm^2^ for HMDSO; for OMCTS, volume of pressure (Vp) = 100 μL, Vp/S = 3.8 mm; for TTMS, Vp = 60 μL, Vp/S = 2.3 mm. For HMDSO, plasma treatment time (Δt) =10 min, number of cycles (N) = 10; for OMCTS and TTMS, Δt = 5 min, N = 1 [73]. Reproduced with permission from Ref. [73]. Copyright 2017, Elsevier.

The synthesis process of functional films onto substrates directly from the liquid phase plays an important role in coating technologies. This process offers the prospects for the deposition of thin films based on the structures of the liquid phase used in the synthesis process. For example, a method based on initializing the synthesis using an “atmospheric pressure plasma jet” running with argon over a thin liquid layer of the starting material was reported by Jan Schäfer et al. [73]. In this synthesis, a plasma-enhanced chemical vapor deposition (PECVD) process is employed for the formation of organosilicon films using the liquid compounds of hexamethyldisiloxane (HMDSO), octamethyltetrasiloxane (OMCTS), and tetrakis(trimethylsilyloxy)silane (TTMS). The schematic illustration of this process along with the applied process parameters is displayed in Figure 5. 

According to the authors, following the plasma procedure, a constant internal polymerization was made possible by the molecular network‘s stability [73]. This process‘s primary benefit is its ability to work with colloids or liquids that have a lower vapor pressure than what is required for vapor transport in PECVD setups. The disadvantage of this approach owing to a relatively high amount of interstitial water that was initially introduced into the films is post-treatment, such as annealing, that is required to improve the film characteristics. As a result, LA-PECVD is a technique that shows promise and opens up new avenues for thin film technology applications. Thus, the interaction of “atmospheric pressure plasma” and a bulk liquid precursor is the force in “atmospheric pressure plasma” synthesis techniques employing liquid-type precursors. The numerous complex chemical and physical reactions at the plasma–liquid interface reason for the reduction, oxidation, and sputtering process. It is important to mention that nanoparticle-like materials are mostly prepared from various radicals produced by the plasma–liquid reactions [38]. There are two categories for this procedure based on where the “atmospheric pressure plasma” is generated. These two types, namely, outside and inside bulk liquid precursors for plasma generation, are reviewed in the following subsections.

#### 2.2.1. Plasma Generated by the Outside Bulk Liquid Precursor

“Atmospheric Pressure Plasma” produced outside of the bulk liquid precursor is also called on-solution plasma influenced by the natural air. The integration between the “Atmospheric Pressure Plasma” and different components of air like O_3_, N_2_O, H_2_, etc. can occur at the plasma–liquid interface [28]. The generation of on-solution plasma using different methods is reported in Refs. [74,75,76]. For example, Tan et al. [74] utilized an “Atmospheric Pressure Plasma Jet” and tungsten electrode for this process using Ar as a discharge gas. In this case, MMA is the liquid precursor where the “Atmospheric Pressure Plasma” deals with the surface of styrene [74]. To activate the “Atmospheric Pressure Plasma”, an AC power source is used from a neon sign transformer at a voltage of 15 kV and a frequency of 60 Hz.

The metal nanoparticles introduced by a conducting polymer were synthesized by Zhang et al. [75] where the authors used the stainless steel capillary and a carbon rod as the electrodes and the liquid surface. Such capillary electrode plays a role in the “Atmospheric Pressure Plasma Jet” for the generation of the He plasma. For this synthesis, the liquid precursor was an aqueous solution of HauCl_4_, which was added to poly(3,4-ethylenedioxy thio- phene) polystyrene sulfonate (PEDOT:PSS). The “Atmospheric Pressure Plasma” was exploded by direct current (DC) maintaining a voltage of 0.8 kV [75]. To synthesize nanographene, a pin-type Cu electrode was employed for the production of on-solution plasma where C_2_H_5_OH acted as a precursor, as in Ref. [76].

In brief, polymer synthesis using liquid-type precursors has numerous particularities reported by researchers [3]. The authors utilized thin solid films, plasma-treated styrene and MMA, nanographene, nanoparticle-incorporated conducting polymer, etc. For this type of synthesis, the employed precursors are styrene, HMDSO, OMCTS), TTMS, HAuCl4, PEDOT:PSS, and C_2_H_5_OH. To generate plasma an AC/DC power source was applied at a voltage of 15 kV and a frequency of 60 Hz to 1.5 MHz. Thus, designing appropriate precursors and generating suitable plasma sources can improve such polymerization process.

#### 2.2.2. Plasma Generated by the Inside Bulk Liquid Precursor

This method is also known as in-solution plasma where the “Atmospheric Pressure Plasma” is generated in liquid precursors. The key point of this technique is the gap between two electrodes. This gap induces the breakdown of plasma in liquid media [77,78]. The tungsten electrode employs an in-solution plasma approach owing to its numerous advantages, such as high melting point and stability, anticorrosion property, and high electrical conductivity [79]. This in-solution plasma approach is mainly utilized by researchers for the fabrication of nanoparticles [80,81,82,83,84]. Other than nanoparticle synthesis, liquid precursors are also used to synthesize nitrogen–carbon nanosheets [80]. In these cases, pyrrolidine, pyrrole, N-methyl-2-pyrrolidone, 2-pyrrolidone, 1-methylpyrrolidine, cyclopentanone, and cyclohexanone were utilized [80]. In most of the cases, researchers utilized a bipolar pulse with an amplitude in the range of 1.7–16.4 kV and a frequency of 5–200 kHz to produce in-solution plasma in the Ar bubble outlet, and the bipolar pulse duty ratio was 1–100 μs [80,81,82,83,84].

In summary, polymer synthesis using liquid-type precursors has significance and particularity, as mentioned in the literature, that depend on the used precursor and plasma source. The significance and advantages include the potential plasma treatment of MMA and styrene, incorporation of metallic NPs on conducting polymers, formation of thin and solid SiOx films, and so on. The utilized precursors for these purposes are styrene, MMA, HMDSO, HAuCl_4_, PEDOT:PSS, C_2_H_5_OH, etc. The plasma sources used for these processes are Ar and He, and the plasma is generated with the use of AC/DC pulse power of 5–450 W with a voltage of 2–15 kV and at a frequency of 60 Hz–1.1 MHz. Thus, more focus can be provided on the utilization of appropriate precursors, suitable plasma sources, and the overall technique of the polymerization process to achieve large-scale industrial applications. 

### 2.3. Polymer Synthesis Using Plasma Techniques

**Figure 6:**
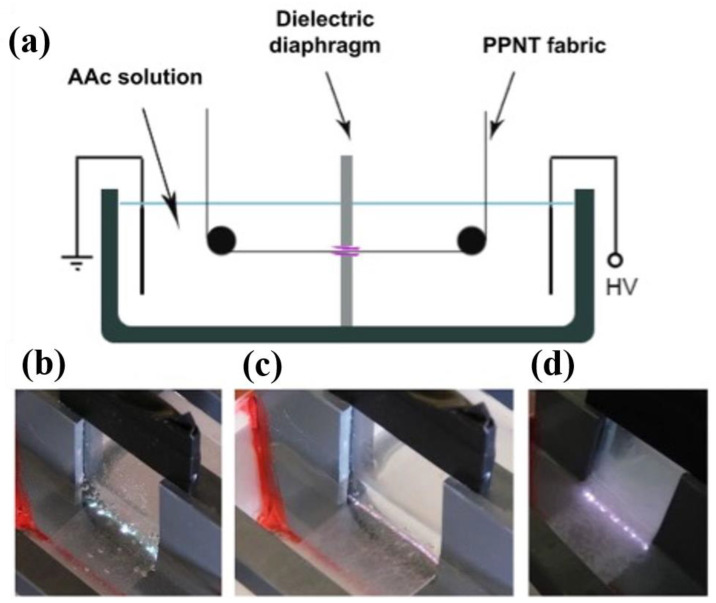
(**a**) A Schematic illustration of the experimental setup of diaphragm discharge; image of plasma generated in the (**b**) tap water, (**c**) 1% NaCl, and (**d**) 20% AAc solution. Permission from the Ref. [85]. Copyright 2014, Springer.

As the multistep preparation is time-consuming and complex in many cases, polymer synthesis using plasma techniques is very useful in those circumstances. For instance, acrylic acid (AAc) is a tremendous origin of carboxylic-rich (-COOH) coatings for polypropylene (PP), which are appropriate for this issue, but their multistep fabrication process is slow. In this case, plasma polymerization offers an exceptional solution to this challenging process since the polymerization approach and grafting to the substrate occur concurrently, as reported by Dušan Kováčik et al. [85]. The authors suggested a comparatively fast and efficient technique for AAc plasma polymerization, utilizing a pulsed immersed diaphragm electrical discharge that worked in an aqueous solution of AAc. The AAc layers were effectively grafted onto PP nonwoven, which are always rewound over the slit [85]. For this purpose, the experimental setup of the diaphragm discharge reactor for the treatment of plasma is presented schematically in Figure 6a.

The parameters of this experiment were as follows: 100 Hz, 25 kV, 75 ns, and 400 ns for the half-width of the pulse, peak voltage, rising time, and frequency [85]. Figure 6b–d represents the image that depicts how the discharge appears in tap water, NaCl solution, and AAc solution, respectively. In conclusion, the ability to execute grafting of AAc to fibrous substrate and plasma-initiated polymerization in the continuous regime presents opportunities for the use of this modified material in biomedical applications. Asandulesa et al. [86] revealed a polymerization mechanism related to this plasma polymerization approach by characterizing “Atmospheric Pressure Plasma”-polymerized films that were synthesized from different monomers and determining the chemical structures of polymer films. Such kind of chemical structures depend on the plasma settings that were employed, for example, in the case of polymerized films based on benzaldehyde [86].

In addition, plasma polymerization follows complex physicochemical reactions that are different from the usual polymerization approach. The high cross-linking feature of plasma polymers is easily created by continuous fragmentation and random recombination, in contrast to traditional chemical polymerization techniques, such as self-assembly, layer-by-layer, or spin coating [87]. Enhanced mechanical stability and fewer morphological modifications can be carried out through the excessive cross-linking ability of plasma polymers [54,88,89].

The economic impact of the numerous synthesis methods of plasma polymerization is a vital factor to be considered. The methods of plasma polymerization are regarded as a green process as the approaches require less amount of chemicals than the conventional synthesis approach and generate a minimum amount of waste products. So, these methods do not require an additional environmental remediation process. These green aspects of polymer synthesis have become significant in the sense of the cost of environmental remediation, which is less expensive than the conventional synthesis approach. The following aspects can be considered for the reported numerous plasma polymerization methods: (i) green processing; (ii) the synthesis process requires fewer precursors; (iii) the process requires no additional environmental remediation process; and (iv) coating based on plasma polymerization can be attained with a low production cost. Besides the economic impact, the yield of plasma-based polymers is also an important factor for large-scale applications. The deposition yield of plasma-based polymers depends on the operational parameters of the synthesis process even if the same monomers and reactors are used for the polymerization process. For instance, Park et al. [90] reported that the specific energy per mass is the vital controlling parameter for the yield of deposition in the case of plasma polymerization of hexamethyldisilazane. The authors also mentioned that such yield differs from the conventional polymer materials obtained from the synthesis approach. Thus, to attain a higher yield of plasma-based polymers, focus can be given to the controlling operational parameters of the synthesis process wherever the synthesis approach is followed.

## 3. Morphological Features

**Figure 7:**
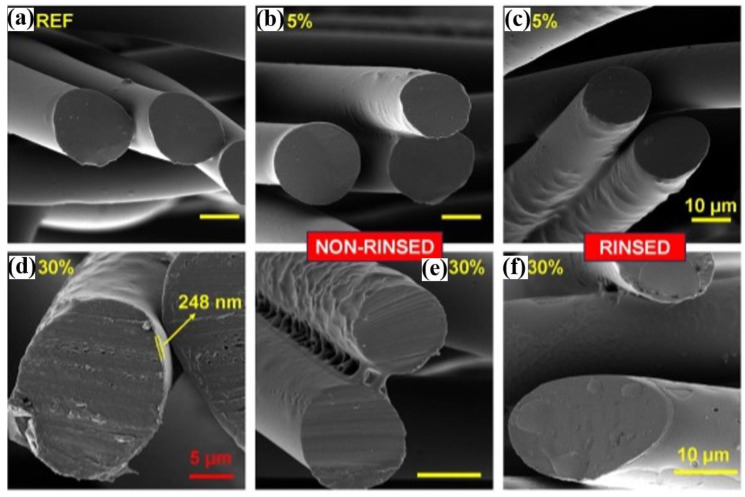
SEM micrograph of (**a**) reference PPNT, (**b**) ppAAc layer from a 5% solution (AAc solution in deionized water) (non-rinsed), (**c**) ppAAc layer from a 5% solution (rinsed), (**d**) ppAAc layer from a 30% solution (AAc solution in deionized water) after 2 rinsing cycles with marked thickness of the layer, (**e**) ppAAc layer from a 30% solution (non-rinsed), and (**f**) ppAAc layer from a 30% solution (rinsed) [85]. Permission from the Ref. [85]. Copyright 2014, Springer.

The morphological features of the polymeric materials obtained by employing the various synthesis methods are significant owing to their possible applications in the biomedical field. The obtained polymers also possess numerous features, including smooth surfaces, highly porous nonuniform surfaces, and so on. For example, the plasma-polymerized (ppAAc) layer, with a smooth surface, attached to the polypropylene nonwoven textile (PPNT) fibers, which are separated, is displayed in Figure 7 [85]. Plasma polymerization results in the vastly porous nonuniform PPNT surface with a higher homogeneity. The authors claimed that the diaphragm discharge-initiated polymerization of acrylic acid is stable even after rinsing and ultrasonic cleaning, as evidenced by the existence of a ppAAc layer on the surface, as presented in Figure 7c,d,f [85]. 

**Figure 8:**
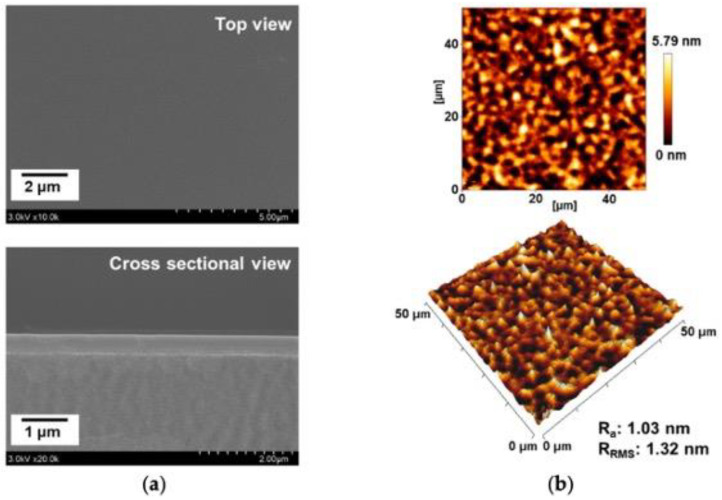
Surface morphology of the PANI film obtained through plasma polymerization process: (**a**) FESEM micrographs of the top and a cross-sectional view of the PANI film; (**b**) two-dimensional and three-dimensional AFM micrographs [92]. Copyright 2021, MDPI.

The formation of PANI films by plasma polymerization with distinct morphological features is reported by G. J. Cruz et al. [91], as shown in Figure 8a,b. The plain morphology of the film is evident in some regions whereas the randomly distributed bubbles with 1–6 mm diameter are visible in the synthesized PANI film (Figure 8a). On the other hand, a continuous surface without any bubbles or discontinuities is seen in the PANI film (Figure 8a). Such morphological characteristics may impact the thermal stability and electrical conductivity of the polymer film obtained through plasma polymerization [91]. J. Y. Kim et al. [91] reported the plasma polymerization of PANI films along with their distinct morphological features. The authors synthesized PANI films on the glass substrate with smooth, homogeneous, and flat morphology having a thickness of 450 nm (Figure 8a). Further, the surface roughness and root mean square roughness values of 1.03 nm and 1.32 nm, respectively, of such PANI film were observed by the authors (Figure 8b) [92].

**Figure 9:**
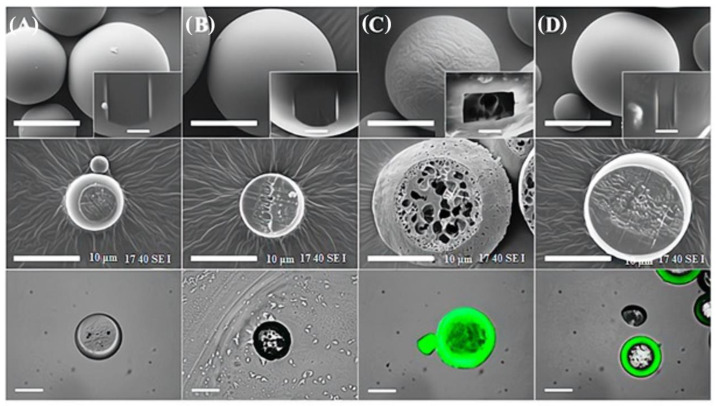
The SEM micrographs depict the PDLLGA microspheres (**A**) at earlier conditions and (**B**) following modification with gel-MA, utilizing distinctive approaches that comprise surface adsorption, (**C**) surface frame-up, and (**D**) oxygen plasma treatment. Cross-sectioned SEM photos show that in entrapment-modified microspheres, the porous structure gets smaller and smaller as it approaches the surface. Each figure is displayed with a 50 μm scale bar with magnified images of 5 μm [93]. Copyright 2023, MDPI.

Therefore, the material‘s surface morphology plays a crucial role in determining its physical and biological characteristics, including surface area, porosity, and roughness [93,94]. For instance, morphology analysis reported by Pankaj Bhatt et al. [94] showed that comparing the internal structures of polymer-based microspheres treated by adsorption and plasma methods with those of non-modified poly(D, L-lactide-co-glycolide) microspheres revealed no appreciable changes. Fluorescence microscopy analysis revealed that cross-sectioned PDLLGA microspheres that were altered by plasma and entrapment techniques had a fluorescent layer on their exteriors. Moreover, internal fluorescent deposition was observed in the entrap-modified microspheres but not in the plasma- or adsorption-modified microspheres [94,95,96]. The SEM micrograph of PDLLGA microspheres is displayed in Figure 9. 

## 4. Biomedical Applications

### 4.1. Biocompatibility Enhancement

The use of plasma is a significant technique for the treatment of polymeric materials to improve their biocompatible properties. Biocompatible properties are influenced by the attainment rate of surface endothelialization, which is highly associated with surface characteristics such as surface wettability, topography, and related phenomena. In this context, plasma treatment is an efficient way to adjust the polymer surface by incorporating functional groups like amine, hydroxyl, and carboxyl groups, etc. Such modification can enhance the respective polymer interactions with cells, drugs, and other related materials [97]. By increasing the surface roughness of the polymer-based nanoparticles utilizing plasma treatments, the cell adhesion properties of the polymers can be improved. In addition, the plasma treatment of the polymeric nanomaterials can enhance their stability by modifying the electrostatic as well as steric stabilization of the concerned polymers [60]. The numerous types of plasma treatment of the polymers as well as their significance and particularity in the context of biocompatibility are listed in Table 1. In summary, plasma treatment of N_2_, O_2_, Ar, radiofrequency (RF) plasma, etc. are used for the treatment of natural and synthetic polymers, as reported by researchers [98,99,100,101,102,103,104]. All of these treatments have some effectiveness in enhancing biocompatibility, as presented in Table 1. However, more focus can be provided on the suitability of the polymers, copolymers, polymer composites, and polymer-based nanoparticles to achieve better performances of biocompatibility.

### 4.2. Uses as Antimicrobial Coatings

Biomedical device-associated infections owing to the microbial colonization of surfaces and subsequent biofilm formation are a vital concern nowadays. Since such infections can originate from surgical tools and other related sources, the treatment process becomes complicated and there may be a need to repeat surgery. In those cases, as plasma polymers can coat a wide variety of materials, they are considered promising for these issues. In this connection, the application of plasma polymers for antibacterial coatings with discussions of the state of the art can be found in the literature [105,106,107].

The deposition of a plasma polymer film like chlorinated organic compounds has an intrinsic antimicrobial property. For example, Michl et al. [108] reported that the plasma deposition of chlorinated hydrocarbons exhibited enhanced antibacterial surface performance against *Staphylococcus epidermidis* depending on the Cl/C ratios. Nitric oxide (NO) is another molecule that can possess biologically important signaling; however, this molecule needs to diffuse into and within biological systems to work properly. Such capability can be attained by plasma polymerization of isopentyl nitrite (C₅H₁₁ONO) [108] against *S. epidermidis* by releasing NO, which can delay bacterial growth without killing adhering bacterial cells. Such bacteriostatic behavior was thought to have potential applications in medicine as a coating for medical equipment that could inhibit the creation of bacterial biofilms [109]. Pegalajar-Jurado et al. [110] applied 1,8-cineol plasma polymer coatings and assessed their efficacy against *Staphylococcus aureus* and *Escherichia coli*. The authors found a substantial decrease in bacterial adhesion for both types of bacteria. 

Al-Jumaili et al. [111] investigated plasma polymer films fabricated on a glass substrate utilizing geranium oil (a mixture of organic compounds) to evaluate antibacterial performance against *S. aureus*, *Pseudomonas aeruginosa*, and *E. coli*. The authors showed that during the colonization of bacteria in plasma polymer coatings, the adherence was lower for the deposited films, utilizing less power (W). This behavior indicates the antiadhesive impact against such type of bacteria [111]. Kumar et al. [112] employed the plasma polymerization of terpinen-4-ol to generate antibacterial surface coatings. The authors conducted an antibacterial experiment exploiting *P. aeruginosa* and observed the existence of dead cells on the coatings with a substantial decrease in live bacteria [112]. Other organic compounds, like diethyl phosphite, were also used by Kaleli-Can et al. [112] for the deposition of the plasma polymer coating. Another category of plasma polymer coatings that possess inherent anti-biofouling characteristics was developed by Vasilev et al. where the authors have shown that these coatings are based on films that are accumulated from precursors of oxazolines, such as 2-methyl-2-oxazoline and 2-ethyl-2-oxazoline [113,114,115]. Such coatings can reduce biofilm growth by up to 90% compared with glass slides or Thermanox controls [114,116]. This kind of study exhibits the prospect of substantially lessening bacterial adhesion to the surfaces compared with restraint biomaterials for bacterial and fungal pathogens, which are human concerns. The study also assessed the human cell compatibility of plasma-based polymers [109,110,117,118]. 

### 4.3. Uses in Attachment of Mammalian Cells

Plasma polymers bear significant capabilities for their application in the biomedical field. For biomedical applications, attaching and adhering the cells to surfaces like synthesized materials and devices is necessary. In this context, plasma polymers can be an exceptional support surface for the attachment and adherence of feasible cells and tissue [119]. Plasma polymers can also afford exceptional surfaces of cell attachment by developing scaffold forms useful for tissue engineering and regenerative medicine. Further, double plasma polymerization is able to create gradients of hydrophilicity and enhance cell adhesion ability, which can increase the rate of cell colonization [120,121,122]. Despite several advantages of plasma polymerization in biomedical applications, there are still challenges in the engineering of plasma processes to adapt to complex 3-D geometries. In this regard, some of the challenges have already been addressed by researchers [121,122,123,124,125,126]. So, more focus and effort are needed to overcome the challenges of using plasma polymerizations for more compatible applications in biomedical sectors.

### 4.4. Silver Coating Using Plasma-Based Polymer

**Figure 10:**
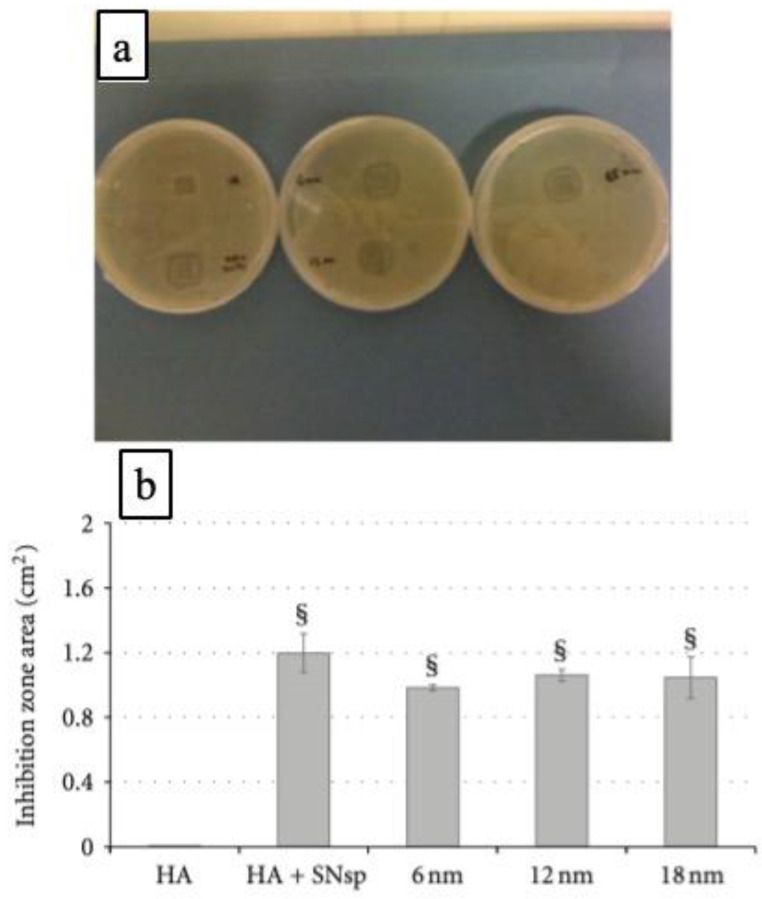
(**a**) An example of the inhibition area identified in the surrounding of the HA film (“HA”), incorporated with SNPs (“HA + SNPs”), and recuperated by a second HA layer of three different thicknesses of 6 nm, 12 nm, and 18 nm (noted that two different samples were positioned on each plate). (**b**) Quantitative outcomes of diffusion test on agar plates for five different kinds of materials. § symbol specifies a major difference compared with HA (*p* < 0.01) [125]. Permission from the reference [127]. Reproduced with permission from Ref. [127]. Copyright 2012, Wiley.

Plasma polymers have attracted interest in investigating the release of silver ions and nano/biocomposite coatings for intended biomedical applications. For example, silver nanoparticle (SNPs)-containing materials can be enclosed by n-heptylamine (HA) polymer without depriving the antibacterial activity of planktonic bacteria existing in the surrounding nanoparticles [127]. The authors showed the antibacterial effects of the SNPs on adhered bacteria and the number of bacteria was lessened with the thickness of the second layer provided by HA. The authors also established that the antibacterial effect originated from the incorporation of HA with SNPs by diffusion assays on nutritive agar plates. The existence of bacterial growth inhibition zones (IZs) surrounding the materials containing SNPs, as well as the nonexistence of IZs for the SNPs-free HA polymer, is presented in Figure 10a [127]. These outcomes confirm the capabilities of the antibacterial effect of the SNPs-containing HA films against *E. coli* growth. Such an antibacterial effect was not observed without the loading s of SNPs with HA polymer.

Further, the area of IZ is insignificantly wider for the HA + SNPs materials compared with only SNPs-containing materials enclosed with a second HA layer (Figure 10b). In addition, IZ area assessments (Figure 10b) show that HA + SNPs material led to an inconsiderably wider IZ than SNPs-containing materials enclosed with a second HA layer. These results indicate that antibacterial activity from Ag^+^ produced by implanted SNPs can occur even in the presence of an 18 nm-thick HA layer [127]. Thus, such a study indicates the importance of the antibacterial activity of silver coating materials utilizing plasma-based polymer.

Owing to the great antibacterial properties of silver-containing materials, they have potential applications in medical devices and industrial sectors [128,129,130]. These silver-containing materials can kill both Gram-positive and Gram-negative bacteria and fungi. Due to the ability of silver to target multiple sites of the bacterial cells, it is much harder for bacteria to show resistance against silver than against common types of antibiotics [108]. Thus, the silver-comprising coatings motivated the plasma polymer-based approaches to contribute to areas of commercial and even clinical purposes [105,107,131]. So, it is hard to cover all aspects of this context by describing each report in this subsection. The intention is rather to provide a taste of the promising probabilities presented by plasma-based polymers.

## 5. Summary and Future Perspective

There are many advantages of plasma polymerization for synthesizing plasma-based polymeric materials, including quicker processing times, reduced expenses, environmentally friendly waste, and so on. The present review describes the topical studies on the synthesis of polymers utilizing various plasma-based approaches. The synthesis techniques are also sorted according to the state of the utilized precursor, such as the gas/aerosol phase or liquid phase. Polymer synthesis utilizing plasma techniques is convenient and can avoid the usual time-consuming multistep preparation process. Thus, plasma polymerization provides a beneficial solution to overcoming a challenging conventional polymerization process. Distinct morphological features of polymeric materials like surface topography attained through the plasma polymerization process impact the biomedical field’s applications. Such possibilities and already reported information are briefly discussed in this review. The utilization of plasma is an excellent technique for treating polymers to enhance biocompatibility, antimicrobial coatings, and silver coating, which is also described in this review. The aim is to provide a perception of plasma-based polymers for biomedical applications. It is anticipated that in the future, different methods for polymer synthesis will serve as models for industrial and large-scale applications while preserving the environment. The appropriate plasma-based synthesis methods to attain desirable surface morphology and the enchantment of their biocompatibility for biomedical applications is still a challenging area of research. Thus, it is noteworthy to perform further investigations of the design and synthesis of polymeric materials utilizing the plasma polymerization process to overcome these challenges and ensure large-scale applications in biomedical sectors. More focus should be provided on the plasma processing for biomaterial coatings necessary for biomedical functions. Thus, research should concentrate on improving plasma sources and technologies, especially the optimization of plasma process parameters and further investigation of polymer-based biocompatibility strategies to improve the field of biomedical applications.

## Figures and Tables

**Table 1:** Numerous polymers, the plasma treatment used, and their significance and particularity are listed.

Polymer	Plasma Treatment	Significance and Particularity	Ref.
Starch in film form	N_2_ plasma	Enhanced cell adhesion	[98]
Cellulose in nanocrystal form	O_2_ plasma	Enhanced cell viability	[99]
Polycarbonate (coated with diamond-like carbon film)	Radiofrequency (RF) plasma	Inhibition of protein adsorption, less platelet adhesion	[100]
Poly(dimethylsiloxane) (coated with diamond-like carbon film)	Ar plasma	Cellular responses in mammalian cells.	[101]
Pectin hydrogels	Ar plasma	Enhanced cell adhesion	[102]
Low-density polyethylene	Plasma source ion implantation	Enhanced surface hydrophobic features	[103]
Polymerized nanoparticles	Reactive gas emissions plasma	Considerably lessened tumor growth in mice	[104]

## References

[1] Langmuir I. (1928). Oscillations in ionized gases. Proc. Natl. Acad. Sci. USA.

[2] Khelifa F., Ershov S., Habibi Y., Snyders R., Dubois P. (2016). Free-radical-induced grafting from plasma polymer surfaces. Chem. Rev..

[3] Jang H.J., Jung E.Y., Parsons T., Tae H.-S., Park C.-S. (2021). A review of plasma synthesis methods for polymer films and nanoparticles under atmospheric pressure conditions. Polymers.

[4] Weltmann K.D., Kolb J.F., Holub M., Uhrlandt D., Simek M., Ostrikov K., Hamaguchi S., Cvelbar U., Cernak M., Locke B. (2019). The future for plasma science and technology. Plasma. Process. Polym..

[5] Zhou H.P., Ye X., Huang W., Wu M.Q., Mao L.N., Yu B., Xu S., Levchenko I., Bazaka K. (2019). Wearable, Flexible, Disposable Plasma-Reduced graphene oxide stress sensors for monitoring activities in austere environments. ACS Appl. Mater. Interfaces.

[6] Levchenko I., Bazaka K., Belmonte T., Keidar M., Xu S. (2018). Advanced materials for next-generation spacecraft. Adv. Mater..

[7] Levchenko I., Xu S., Wu Y.-L., Bazaka K. (2020). Hopes and concerns for astronomy of satellite constellations. Nat. Astron..

[8] Levchenko I., Baranov O., Fang J., Cherkun O., Xu S., Bazaka K. (2021). Focusing plasma jets to achieve high current density: Feasibility and opportunities for applications in debris removal and space exploration. Aerosp. Sci. Technol..

[9] Coad B.R., Favia P., Vasilev K., Griesser H.J. (2022). Plasma polymerization for biomedical applications: A review. Plasma Process Polym..

[10] Jorg F.F. (2021). Plasma Polymerization. The Plasma Chemistry of Polymer Surfaces: Advanced Techniques for Surface Design.

[11] Goodman J. (1960). The formation of thin polymer films in the gas discharge. J. Polym. Sci..

[12] Lawton E.L. (1974). Adhesion improvement of tire cord induced by gas plasma. J. Appl. Polym. Sci..

[13] Stille J.K., Rix C.E. (1966). The reaction of halobenzenes in a radiofrequency glow discharge. J. Org. Chem..

[14] Denaro A.R., Owens P.A., Crawshaw A. (1969). Glow discharge polymerization-II α-methylstyrene, ω-methylstyrene and allylbenzene. Eur. Polym. J..

[15] Kobayashi H., Bell A.T., Shen M. (1973). Formation of an amorphous powder during the polymerization of ethylene in a radio-frequency discharge. J. Appl. Polym. Sci..

[16] Coad B.R., Scholz T., Vasilev K., Hayball J.D., Short R.D., Griesser H.J. (2012). Functionality of proteins bound to plasma polymer surfaces. ACS Appl. Mater. Interfaces..

[17] Truica-Marasescu F., Wertheimer M.R. (2008). Nitrogen-rich plasma-polymer films for biomedical applications. Plasma Process. Polym..

[18] Vasani R.B., Szili E.J., Rajeev G., Voelcker N.H. (2017). On-demand antimicrobial treatment with antibiotic-loaded porous silicon capped with a pH-responsive dual plasma polymer barrier. Chem. Asian J..

[19] Seo H.J., Gil Y.E., Hwang K.-H., Ananth A., Boo J.-H. (2019). Synthesis and characterization of plasma-polymer gate dielectric films for graphene field effect transistor devices. Electron. Mater. Lett..

[20] Inagaki N., Tasaka S., Ikeda Y. (1995). Plasma polymerization of copper phthalocyanines and application of the plasma polymer films to NO_2_ gas sensor device. J. Appl. Polym. Sci..

[21] He J.-H., Singamaneni S., Ho C.H., Lin Y.-H., McConney M.E., Tsukruk V.V. (2009). A thermal sensor and switch based on a plasma polymer/ZnO suspended nanobelt bimorph structure. Nanotechnology.

[22] Bhatt S., Pulpytel J., Aref-Khonsari F. (2015). Low and atmospheric plasma polymerisation of nanocoatings for bio-applications. Surf. Innov..

[23] Hegemann D., Lorusso E., Butron-Garcia M.-I., Blanchard N.E., Rupper P., Favia P., Heuberger M., Vandenbossche M. (2016). Suppression of hydrophobic recovery by plasma polymer films with vertical chemical gradients. Langmuir.

[24] Rao J., Bao L., Wang B., Fan M., Feo L. (2018). Plasma surface modification and bonding enhancement for bamboo composites. Compos. Part B Eng..

[25] Ishijima T., Nosaka K., Tanaka Y., Uesugi Y., Goto Y., Horibe H. (2013). A high-speed photoresist removal process using multibubble microwave plasma under a mixture of multiphase plasma environment. Appl. Phys. Lett..

[26] Bitar R., Cools P., Geyter N.D., Morent R. (2018). Acrylic acid plasma polymerization for biomedical use. Appl. Surf. Sci..

[27] Moreau M., Orange N., Feuilloley M.G.J. (2008). Non-thermal plasma technologies: New tools for bio-decontamination. Biotechnol. Adv..

[28] Mun M.K., Lee W.O., Park J.W., Kim D.S., Yeom G.Y., Kim D.W. (2017). Nanoparticles synthesis and modification using solution plasma process. Appl. Sci. Converg. Technol..

[29] Xu Y., Dai L., Chen J., Gal J.-Y., Wu H. (2007). Synthesis and characterization of aniline and aniline-o-sulfonic acid copolymers. Eur. Polym. J..

[30] Mariotti D., Sankaran R.M. (2010). Microplasmas for nanomaterials synthesis. J. Phys. D Appl. Phys..

[31] Özçiçek N.P., Pekmez K., Holze R., Yildiz A. (2003). Spectroelectrochemical investigations of aniline-thiophene copolymers in acetonitrile. J. Appl. Polym. Sci..

[32] Muzammil I., Li Y., Lei M. (2017). Tunable wettability and pH-responsiveness of plasma copolymers of acrylic acid and octafluorocy-clobutane. Plasma Process. Polym..

[33] Liu T., Yang F., Li Y., Ren L., Zhang L., Xu K., Wang X., Xu C., Gao J. (2014). Plasma synthesis of carbon nanotube-gold nanohybrids: Efficient catalysts for green oxidation of silanes in water. J. Mater. Chem. A..

[34] Fauchais P., Etchart-Salas R., Rat V., Coudert J.F., Caron N., Wittmann-Ténèze K. (2008). Parameters controlling liquid plasma spraying: Solutions, sols, or suspensions. J. Therm. Spray Technol..

[35] Yang P., Zhang J., Guo Y. (2009). Synthesis of intrinsic fluorescent polypyrrole nanoparticles by atmospheric pressure plasmapolymerization. Appl. Surf. Sci..

[36] Teslaru T., Topala I., Dobromir M., Pohoata V., Curecheriu L., Dumitrascu N. (2016). Polythiophene films obtained by polymerization under atmospheric pressure plasma conditions. Mater. Chem. Phys..

[37] Saito G., Akiyama T. (2015). Nanomaterial synthesis using plasma generation in liquid. J. Nanomater..

[38] Rezaei F., Vanraes P., Nikiforov A., Morent R., Geyter N.D. (2019). Applications of plasma-liquid systems: A review. Materials.

[39] Seebock R., Esrom H., Charbonnier M., Romand M., Kogelschatz U. (2001). Surface modification of polyimide using dielectric barrier discharge treatment. Surf. Coat. Technol..

[40] Mittal K.L. (1976). Adhesion aspects of metallization of organic polymer surfaces. J. Vac. Sci. Technol..

[41] Zhang P., Zhang S., Kong F., Zhang C., Dong P., Yan P., Cheng X., Ostrikov K.K., Shao T. (2020). Atmospheric-pressure plasma jet deposition of bumpy coating improves polypropylene surface flashover performance in vacuum. Surf. Coat. Technol..

[42] Pandiyaraj K.N., Ramkumar M.C., Kumar A.A., Vasu D., Padmanabhan P.V.A., Tabaei P.S.E., Cools P., Geyter N.D., Morent R., Jaganathan S.K. (2019). Development of phosphor containing functional coatings via cold atmospheric pressure plasma jet—Study of various operating parameters. Appl. Surf. Sci..

[43] Hossain M.M., Trinh Q.H., Nguyen D.B., Sudhakaran M.S.P., Mok Y.S. (2019). Formation of plasma-polymerized superhydrophobic coating using an atmospheric-pressure plasma jet. Thin Solid Film..

[44] Jang H.J., Park C.-S., Jung E.Y., Bae G.T., Shin B.J., Tae H.-S. (2020). Synthesis and properties of thiophene and aniline copolymer using atmospheric pressure plasma jets copolymerization technique. Polymers.

[45] Park C.-S., Jung E.Y., Jang H.J., Bae G.T., Shin B.J., Tae H.-S. (2019). Synthesis and properties of plasma-polymerized methyl methacrylate via the atmospheric pressure plasma polymerization technique. Polymers.

[46] Doherty K.G., Oh J.S., Unsworth P., Sheridan C.M., Weightman P., Bradley J.W., Williams R.L. (2019). Plasma polymerization using helium atmospheric-pressure plasma jet with heptylamine monomer. Plasma Process. Polym..

[47] Kim J.Y., Iqbal S., Jang H.J., Jung E.Y., Bae G.T., Park C.-S., Tae H.-S. (2021). In-situ iodine doping characteristics of conductive polyaniline film polymerized by low-voltage-driven atmospheric pressure plasma. Polymers.

[48] Yan X., Liu G.-S., Yang J., Pu Y., Chen S., He H.-W., Wang C., Long Y.-Z., Jiang S. (2019). In situ surface modification of paper-based relics with atmospheric pressure plasma treatment for preservation purposes. Polymers.

[49] Karl C.W., Rahimi W., Kubowicz S., Lang A., Geisler H., Giese U. (2020). Surface modification of ethylene propylene diene terpolymer rubber by plasma polymerization using organosilicon precursors. ACS Appl. Polym. Mater..

[50] Yang J., Pu Y., Miao D., Ning X. (2018). Fabrication of durably superhydrophobic cotton fabrics by atmospheric pressure plasma treatment with a siloxane precursor. Polymers.

[51] Mertens J., Nisol B., Hubert J., Reniers F. (2020). Use of remote atmospheric mass spectrometry in atmospheric plasma polymerization of hydrophilic and hydrophobic coatings. Plasma Process. Polym..

[52] Getnet T.G., da Silva G.F., Duarte I.S., Kayama M.E., Rangel E.C., Cruz N.C. (2020). Atmospheric pressure plasma chemical vapor deposition of carvacrol thin films on stainless steel to reduce the formation of *E. coli* and *S. aureus* biofilms. Materials.

[53] Sťahel P., Mazánková V., Tomečková K., Matoušková P., Brablec A., Prokeš L., Jurmanová J., Buršíková V., Přibyl R., Lehocký M. (2019). Atmospheric pressure plasma polymerized oxazoline-based thin films-antibacterial properties and cytocompatibility performance. Polymers.

[54] Bardon J., Martin A., Fioux P., Amari T., Mertz G., Delmée M., Ruch D., Roucoules V. (2018). Reinforcement of a dodecylacrylate plasma polymer by admixture of a diacrylate or a dimethacrylate cross-linker. Plasma Process. Polym..

[55] Demaude A., Poleunis C., Goormaghtigh E., Viville P., Lazzaroni R., Delcorte A., Gordon M., Reniers F. (2019). Atmospheric pressure plasma deposition of hydrophilic/phobic patterns and thin film laminates on any surface. Langmuir.

[56] Ma C., Wang L., Nikiforov A., Onyshchenko Y., Cools P., Ostrikov K., Geyter N.D., Morent R. (2021). Atmospheric-pressure plasma assisted engineering of polymer surfaces: From high hydrophobicity to superhydrophilicity. Appl. Surf. Sci..

[57] Loyer F., Bengasi G., Frache G., Choquet P., Boscher N.D. (2018). Insights in the initiation and termination of poly (alkyl acrylates) synthesized by atmospheric pressure plasma-initiated chemical vapor deposition (AP-PiCVD). Plasma Process. Polym..

[58] Loyer F., Combrisson A., Omer K., Moreno-Couranjou M., Choquet P., Boscher N.D. (2019). Thermoresponsive water-solublepolymer layers and water-stable copolymer layers synthesized by atmospheric plasma initiated chemical vapor deposition. ACS Appl. Mater. Interfaces.

[59] Abessolo Ondo D., Loyer F., Werner F., Leturcq R., Dale P.J., Boscher N.D. (2019). Atmospheric-pressure synthesis of atomically smooth, conformal, and ultrathin low-k polymer insulating layers by plasma-initiated chemical vapor deposition. ACS Appl. Polym. Mater..

[60] Khoo Y.S., Lau W.J., Liang Y.Y., Karaman M., Gürsoy M., Lai G.S., Ismail A.F. (2021). Rapid and eco-friendly technique for surface modification of TFC RO membrane for improved filtration performance. J. Environ. Chem. Eng..

[61] Kim D.H., Kim H.J., Park C.-S., Shin B.J., Seo J.H., Tae H.-S. (2015). Atmospheric pressure plasma polymerization using double grounded electrodes with He/Ar mixture. AIP Adv..

[62] Kodaira F.V.P., Ricci Castro A.H., Prysiazhnyi V., Mota R.P., Quade A., Kostov K.G. (2017). Characterization of plasma polymerized HMDSN films deposited by atmospheric plasma jet. Surf. Coat. Technol..

[63] Malinowski S., Herbert P.A.F., Rogalski J., Jaroszyńska-Wolińska J. (2018). Laccase enzyme polymerization by soft plasma jet for durable bioactive coatings. Polymers.

[64] Park C.-S., Kim D.Y., Kim D.H., Lee H.-K., Shin B.J., Tae H.-S. (2017). Humidity-independent conducting polyaniline films synthe sized using advanced atmospheric pressure plasma polymerization with in-situ iodine doping. Appl. Phys. Lett..

[65] Kim D.H., Park C.-S., Kim W.H., Shin B.J., Hong J.G., Park T.S., Seo J.H., Tae H.-S. (2017). Influences of guide-tube and bluff-body on advanced atmospheric pressure plasma source for single-crystalline polymer nanoparticle synthesis at low temperature. Phys. Plasmas.

[66] Moosburger-Will J., Bauer M., Schubert F., Kunzmann C., Lachner E., Zeininger H., Maleika M., Hönisch B., Küpfer J., Zschoerper N. (2017). Methyltrimethoxysilane plasma polymerization coating of carbon fiber surfaces. Surf. Coat. Technol..

[67] Ibrahim J., Al-Babtaineh S.A., Cousens S., Michelmore A., Corr C., Whittle J. (2021). A surface dielectric barrier discharge for deposition of allylamine polymer costings. Appl. Surf. Sci..

[68] Ramkumar M.C., Navaneetha Pandiyaraj K., Arun Kumar A., Padmanabhan P.V.A., Cools P., Geyter N.D., Morent R., Uday Kumar S., Kumar V., Gopinath P. (2017). Atmospheric pressure non-thermal plasma assisted polymerization of poly (ethylene glycol) methylether methacrylate (PEGMA) on low density polyethylene (LDPE) films for enhancement of biocompatibility. Surf. Coat. Technol..

[69] Pandiyaraj K.N., Ramkumar M.C., Arun Kumar A., Padmanabhan P.V.A., Pichumani M., Bendavid A., Cools P., Geyter N.D., Morent R., Kumar V. (2019). Evaluation of surface properties of low-density polyethylene (LDPE) films tailored by atmospheric pressure non-thermal plasma (APNTP) assisted co-polymerization and immobilization of chitosan for improvement of antifouling properties. Mater. Sci. Eng. C.

[70] Dvořáková H., Čech J., Stupavská M., Prokeš L., Jurmanová J., Buršíková V., Ráheľ J., Sťahel P. (2019). Fast surface hydrophilization via atmospheric pressure plasma polymerization for biological and technical applications. Polymers.

[71] Manakhov A., Michlícek M., Necas D., Polcák J., Makhneva E., Eliáš M., Zajíčková L. (2016). Carboxyl-rich coatings deposited by atmospheric plasma co-polymerization of maleic anhydride and acetylene. Surf. Coat. Technol..

[72] Jalaber V., Del Frari D., De Winter J., Mehennaoui K., Planchon S., Choquet P., Detrembleur C., Moreno-Couranjou M. (2019). Atmospheric aerosol assisted pulsed plasma polymerization: An environmentally friendly technique for tunable catechol-bearing thin films. Front. Chem..

[73] Schäfer J., Fricke K., Mika F., Pokorná Z., Zajíčková L., Foest R. (2017). Liquid assisted plasma enhanced chemical vapour deposition with a non-thermal plasma jet at atmospheric pressure. Thin Solid Film..

[74] Tan P.E.C., Mahinay C.L.S., Culaba I.B., Streeter O.K.M., Hilario M.R.A. (2018). Plasma polymerization of styrene using an argon-fed atmospheric pressure plasma jet. J. Vac. Sci. Technol. B.

[75] Zhang R.-C., Sun D., Zhang R., Lin W.-F., Macias-Montero M., Patel J., Askari S., McDonald C., Mariotti D., Maguire P. (2017). Gold nanoparticle-polymer nanocomposites synthesized by room temperature atmospheric pressure plasma and their potential for fuel cell electrocatalytic application. Sci. Rep..

[76] Gamaleev V., Kajikawa K., Takeda K., Hiramatsu M. (2018). Investigation of nanographene produced by in-liquid plasma for development of highly durable polymer electrolyte fuel cells. J. Carbon Res..

[77] Pech D., Brunet M., Durou H., Huang P., Mochalin V., Gogotsi Y., Taberna P.-L., Simon P. (2010). Ultrahigh-power micrometre-sized supercapacitors based on onion-like carbon. Nat. Nanotechnol..

[78] Omurzak E., Shimokawa W., Taniguchi K., Chen L., Okamoto M., Iwasaki H., Yamasaki M., Kawamura Y., Sulaimankulova S., Mashimo T. (2011). Synthesis of wurtzite-type ZnMgS by the pulsed plasma in liquid. Jpn. J. Appl. Phys..

[79] Horikoshi S., Serpone N. (2017). In-liquid plasma: A novel tool in the fabrication of nanomaterials and in the treatment of wastewaters. RSC Adv..

[80] Hyun K.Y., Ueno T., Li O.L., Saito N. (2016). Synthesis of heteroatom-carbon nanosheets by solution plasma processing using N-methyl-2-pyrrolidone as precursor. RSC Adv..

[81] Lee S., Heo Y.K., Bratescu M.A., Ueno T., Saito N. (2017). Solution plasma synthesis of a boron-carbon-nitrogen catalyst with a controllable bond structure. Phys. Chem. Chem. Phys..

[82] Tipplook M., Pornaroontham P., Watthanaphanit A., Saito N. (2020). Liquid-phase plasma-assisted in situ synthesis of amino-rich nanocarbon for transition metal ion adsorption. ACS Appl. Nano Mater..

[83] Shin J.-G., Park C.-S., Jung E.Y., Shin B.J., Tae H.S. (2019). Synthesis of a polyaniline nanoparticle using a solution plasma process with an Ar gas bubble channel. Polymers.

[84] Shin J.-G., Shin B.J., Jung E.Y., Park C.-S., Kim J.Y., Tae H.-S. (2020). Effects of a dielectric barrier discharge (DBD) on characteristics of polyaniline nanoparticles synthesized by a solution plasma process with an Ar gas bubble channel. Polymers.

[85] Kováčik D., Šrámková P., Multáňová P., Stupavská M., Siadati S., Ďurina P., Zahoranov A. (2024). Plasma-induced polymerization and grafting of acrylic acid on the polypropylene nonwoven fabric using pulsed underwater diaphragm electrical discharge. Plasma Chem. Plasma Process..

[86] Asandulesa M., Topala I., Pohoata V., Legrand Y.M., Dobromir M., Totolin M., Dumitrascu N. (2013). Chemically polymerization mechanism of aromatic compounds under atmospheric pressure plasma conditions. Plasma Chem. Plasma Process..

[87] Vandenbossche M., Hegemann D. (2018). Recent approaches to reduce aging phenomena in oxygen-and nitrogen-containing plasma polymer films: An overview. Curr. Opin. Solid State Mater. Sci..

[88] Hegemann D., Hossain M.M., Korner E., Balazs D.J. (2007). Macroscopic description of plasma polymerization. Plasma Process. Polym..

[89] Ligot S., Bousser E., Cossement D., Klemberg Sapieha J., Viville P., Dubois P., Snyders R. (2015). Correlation between mechanical properties and cross-linking degree of ethyl lactate plasma polymer films. Plasma Process. Polym..

[90] Park S.Y., Kim N., Kim U.Y., Hong S.I., Sasabe H. (1990). Plasma polymerization of hexamethyldisilazane. Polym. J..

[91] Cruz G.J., Morales J., Castillo-Ortega M.M., Olayo R. (1997). Synthesis of polyaniline films by plasma polymerization. Synth. Met..

[92] Kim J.Y., Iqbal S., Jang H.J., Jung E.Y., Bae G.T., Park C.S., Shin B.J., Tae H.S. (2021). Transparent polyaniline thin film synthesized using a low-voltage-driven atmospheric pressure plasma reactor. Materials.

[93] Dang Y., Guan J. (2020). Nanoparticle-Based Drug Delivery Systems for Cancer Therapy. Smart Mater. Med..

[94] Bhatt P., Kumar V., Subramaniyan V., Nagarajan K., Sekar M., Chinni S.V., Ramachawolran G. (2023). Plasma modification techniques for natural polymer-based drug delivery systems. Pharmaceutics.

[95] Baki A., Rahman C.V., White L.J., Scurr D.J., Qutachi O., Shakesheff K.M. (2017). Surface modification of PDLLGA microspheres with gelatine methacrylate: Evaluation of adsorption, entrapment, and oxygen plasma treatment approaches. Acta Biomater..

[96] Lieberman M.A., Lichtenberg A.J. (2005). Principles of Plasma Discharges and Materials Processing.

[97] Farr N., Thanarak J., Schäfer J., Quade A., Claeyssens F., Green N., Rodenburg C. (2021). Understanding surface modifications induced via argon plasma treatment through secondary electron hyperspectral imaging. Adv. Sci..

[98] Ghobeira R., Philips C., Declercq H., Cools P., De Geyter N., Cornelissen R., Morent R. (2017). Effects of different sterilization methods on the physico-chemical and bioresponsive properties of plasma-treated polycaprolactone Films. Biomed. Mater..

[99] Bullard K.K., Srinivasarao M., Gutekunst W.R. (2020). Modification of cellulose nanocrystal surface chemistry with diverse nucleo philes for materials integration. J. Mater. Chem. A.

[100] Yoshida S., Hagiwara K., Hasebe T., Hotta A. (2013). Surface modification of polymers by plasma treatments for the enhancement of biocompatibility and controlled drug release. Surf. Coat. Technol..

[101] Nagashima S., Hasebe T., Tsuya D., Horikoshi T., Ochiai M., Tanigawa S., Koide Y., Hotta A., Suzuki T. (2012). Controlled formation of wrinkled diamond-like carbon (DLC) film on grooved poly(dimethylsiloxane) substrate. Diam. Relat. Mater..

[102] Tan G., Chen R., Ning C., Zhang L., Ruan X., Liao J. (2012). effects of argon plasma treatment on surface characteristic of photopolymerization PEGDA–HEMA hydrogels. J. Appl. Polym. Sci..

[103] Kim Y., Lee Y., Han S., Kim K.-J. (2006). Improvement of hydrophobic properties of polymer surfaces by plasma source ion implantation. Surf. Coat. Technol..

[104] Michael P., Lam Y.T., Filipe E.C., Tan R.P., Chan A.H.P., Lee B.S.L., Feng N., Hung J., Cox T.R., Santos M. (2020). Plasma polymerized nanoparticles effectively deliver dual SiRNA and drug therapy in vivo. Sci. Rep..

[105] Vasilev K., Griesser S.S., Griesser H.J. (2011). Antibacterial surfaces and coatings produced by plasma techniques. Plasma. Process. Polym..

[106] Nikiforov A., Deng X.L., Xiong Q., Cvelbar U., De Geyter N., Morent R., Leys C. (2016). Non-thermal plasma technology for the development of antimicrobial surfaces: A review. J. Phys. D Appl. Phys..

[107] Sardella E., Palumbo F., Camporeale G., Favia P. (2016). Nonequilibrium plasma processing for the preparation of antibacterial surfaces. Materials.

[108] Michl T.D., Coad B.R., Husler A., Valentin J.D.P., Vasilev K., Griesser H.J. (2016). Effects of precursor and deposition conditions on prevention of bacterial biofilm growth on chlorinated plasma polymers. Plasma Process. Polym..

[109] Michl T.D., Coad B.R., Doran M., Osiecki M., Kafshgari M.H., Voelcker N.H., Husler A., Vasilev K., Griesser H.J. (2015). Nitric oxide releasing plasma polymer coating with bacteriostatic properties and no cytotoxic side effects. Chem. Commun..

[110] Pegalajar-Jurado A., Easton C.D., Styan K.E., McArthur S.L. (2014). Antibacterial activity studies of plasma polymerised cineole films. J. Mater. Chem. B.

[111] Al-Jumaili A., Bazaka K., Jacob M.V. (2017). Review on the antimicrobial properties of carbon nanostructures. Nanomaterials.

[112] Kumar A., Al-Jumaili A., Prasad K., Bazaka K., Mulvey P., Warner J., Jacob M.V. (2020). Pulse plasma deposition of Terpinen-4-ol: An insight into polymerization mechanism and enhanced antibacterial response of developed thin films. Plasma Chem. Plasma Process..

[113] Macgregor-Ramiasa M.N., Cavallaro A.A., Vasilev K. (2015). Properties and reactivity of polyoxazoline plasma polymer films. J. Mater. Chem. B.

[114] Ramiasa M., Cavallaro A., Mierczynska A., Christo S., Gleadle J., Hayball J., Vasilev K. (2015). Plasma polymerised polyoxazoline thin films for biomedical applications. Chem. Commun..

[115] Cavallaro A.A., Macgreggor-Ramiasa M.N., Vasilev K.A. (2017). Plasma Polymerised Oxazoline Coatings and Uses Thereof.

[116] Cavallaro A.A., Macgregor-Ramiasa M.N., Vasilev K. (2016). Antibiofouling properties of plasma-deposited oxazoline-based thin films. ACS Appl. Mater. Interfaces.

[117] Chan Y.W., Siow K.S., Ng P.Y., Gires U., Yeop Majlis B. (2016). Plasma polymerized carvone as an antibacterial and biocompatible coating. Mater. Sci. Eng. C Mater. Biol. Appl..

[118] Kaleli-Can G., OOzguzar H.F., Kahriman S., Turkal M., Gocmen J.S., Yurtcu E., Mutlu M. (2020). Improvement in antimicrobial properties of titanium by diethyl phosphite plasma-based surface modification. Mater. Today Commun..

[119] Gombotz W.R., Hoffman A.S. (1987). Gas-discharge techniques for biomaterial modification. Crit. Rev. Biocompat..

[120] Barry J.J.A., Howard D., Shakesheff K.M., Howdle S.M., Alexander M.R. (2006). Using a core-sheath distribution of surfacechemistry through 3d tissue engineering scaffolds to control cell ingress. Adv. Mater..

[121] Intranuovo F., Gristina R., Brun F., Mohammadi S., Ceccone G., Sardella E., Rossi F., Tromba G., Favia P. (2014). Plasma modification of pcl porous scaffolds fabricated by solvent casting/particulate-leaching for tissue engineering. Plasma. Process. Polym..

[122] Sardella E., Salama R.A., Waly G.H., Habib A.N., Favia P., Gristina R. (2017). Improving internal cell colonization of porous scaffolds with chemical gradients produced by plasma assisted approaches. ACS Appl. Mater. Interfaces.

[123] Barry J.J., Silva M.M., Shakesheff K.M., Howdle S.M., Alexander M.R. (2005). Using plasma deposits to promote cell population of the porous interior of three-dimensional Poly (D, L-Lactic Acid) tissue-engineering scaffolds. Adv. Funct. Mater..

[124] Intranuovo F., Sardella E., Gristina R., Nardulli M., White L., Howard D., Shakesheff K.M., Alexander M.R., Favia P. (2011). E-CVD processes improve cell affinity of polymer scaffolds for tissue engineering. Surf. Coat. Technol..

[125] Sardella E., Fisher E.R., Shearer J.C., Garzia Trulli M., Gristina R., Favia P. (2015). N_2_/H_2_O plasma assisted functionalization of poly(ε-caprolactone) porous scaffolds: Acidic/basic character versus cell behavior. Plasma. Process. Polym..

[126] Armenise V., Gristina R., Favia P., Cosmai S., Fracassi F., Sardella E. (2020). Plasma-assisted deposition of magnesium-containing coatings on porous scaffolds for bone tissue engineering. Coatings.

[127] Ploux L., Mateescu M., Anselme K., Vasilev K. (2012). Antibacterial properties of silver-loaded plasma polymer coatings. J. Nanomater..

[128] Vasilev K., Cook J., Griesser H.J. (2009). Antibacterial surfaces for biomedical devices. Expert Rev. Med. Devices.

[129] Chernousova S., Epple M. (2013). Silver as antibacterial agent: Ion, nanoparticle, and metal. Angew. Chem. Int. Ed..

[130] Haidari H., Garg S., Vasilev K., Kopecki Z., Cowin A.J. (2020). Silver-based wound dressings: Current issues and future developments for treating bacterial infections. Wound Pract. Res..

[131] Vasilev K. (2019). Nanoengineered antibacterial coatings and aaterials: A perspective. Coatings.

